# Status of Beekeeping Industry in Tanzania: Resources, Practices, and Conservation

**DOI:** 10.3390/insects17020191

**Published:** 2026-02-11

**Authors:** Ismail Seleman Mussa, Shibonage Kulindwa Mashilingi, Shangning Yang, Huoqing Zheng

**Affiliations:** 1 College of Animal Sciences, Zhejiang University, No. 866 Yuhangtang Road, Hangzhou 310058, China; ismailseleman43@gmail.com (I.S.M.); yangsn@zju.edu.cn (S.Y.); 2 Department of Crop Science and Beekeeping Technology, College of Agriculture and Food Technology, University of Dar es Salaam, Dar es Salaam P.O. Box 35134, Tanzania; kulindwasm@gmail.com

**Keywords:** apiary, beekeeping, bee products, honeybee, Tanzania

## Abstract

Beekeeping is a widespread economic activity in rural Tanzania, supporting over 2 million livelihoods. The country’s forests and woodlands, covering approximately 55% of its land area, provide habitat for an estimated 9.2 million honeybee colonies. This positions Tanzania as the second-largest honey producer in Africa and tenth globally. Absence of current information and effective policies hinders exploitation of the industry’s potential. This review presents scientific insights into Tanzania’s beekeeping sector, focusing on honeybee species, bee products, management practices, and conservation. Among three documented subspecies of *Apis mellifera* (Linnaeus, 1758), *A. m. scutellata* is the most widespread and commonly managed by indigenous beekeepers. Tanzania annually produces over 31,000 tonnes of honey and 1800 tonnes of beeswax, generating approximately USD 77.5 million and contributing about 1% to national GDP. The industry supports livelihoods, food security, and biodiversity conservation. Its sustained growth requires effective legal and administrative support, expanded scientific research, enhanced innovation, coordinated partnerships, and integrated nationwide initiatives.

## Simple Summary

Beekeeping is a recognized agricultural and conservation activity with significant socio-economic and ecological benefits in Tanzania. However, the sector faces various constraints such as policy gaps, limited technology adoption, and insufficient research. This review assesses the current state of beekeeping and identifies priority policies and research needed to build a resilient conservation-oriented industry. By presenting available information, it provides an evidence base for policy makers, researchers, and stakeholders. The goal is to enhance governance, resource allocation, and industry practices, thereby securing the sector’s sustainable contribution to socio-economic development and biodiversity conservation.

## 1. Introduction

Tanzania is rich in arable land and forests that sustain diverse wildlife and bee populations [1]. Approximately 13 subspecies of the western honeybee (*A. mellifera*) are adapted to African ecosystems [2,3]. Indigenous beekeepers in Tanzania utilize three well-documented honeybee subspecies [4]. Government initiatives like establishment of the National Beekeeping Policy (NBP) in 1998 have strengthened the beekeeping sector [5]. Formation of beekeepers’ groups, cooperative associations, community banks, forest reserves, and awareness campaigns has engaged women and youth in beekeeping [6]. These efforts aimed at enhancing production, honey and beeswax export earnings, socio-economic development, and environmental conservation [7]. Beekeeping contributes to Tanzania’s agricultural sector through pollination, generates income from bee products, and supports forest and wildlife conservation [8]. 

Despite the contributions of beekeeping in Tanzania, comprehensive information on its current status remains limited. This review presents recent scientific evidence on honeybee subspecies diversity, forage resources, habitats, beekeeping operations, and the properties and consumption of bee products. It provides updated insights into the sector’s role in socio-economic development and biodiversity conservation. It also offers actionable guidance for conservation planning, strategy development, and resource allocation, and directs researchers and practitioners toward focused priorities for a sustainable industry.

## 2. Governance and Economic Contribution of the Beekeeping Industry

Formal beekeeping operations in Tanzania began in 1949 with the Beekeeping Division under the Ministry of Agriculture, aiming to enhance extension services and promote conservation through honey production [9]. A major shift occurred in 1998 with the National Beekeeping Policy (NBP) that transferred oversight to the Ministry of Natural Resources and Tourism (MNRT) [7]. This led formation of the Forestry and Beekeeping Division (FDB) that mandated to coordinate forestry and beekeeping activities [5]. The sector has grown substantially through strengthened frameworks by the MNRT. In 2024, beekeeping contributed approximately USD 77.5 million annually, about 1% of the national GDP [10]. Export earnings from honey and beeswax were approximately USD 12.9 million in 2021 [10,11]. The industry employed around 2 million people across its value chain from 1998 to 2020, including 1.2 million beekeepers and 800,000 individuals in processing and trade, representing nearly 4% of the national population [5,8,12]. However, outdated statistics constrain effective beekeeping governance and resource allocation. Integrating technology into beekeeping registration systems can improve data availability on beekeepers and sectoral contributions. Enhanced stakeholder coordination and strengthened human resource capacity is essential for improving governance, sustainability, and the industry’s long-term economic impact.

## 3. Honeybee Subspecies, Habitat, and Forage Resources

### 3.1. Honeybee Subspecies Diversity

**Figure 1:**
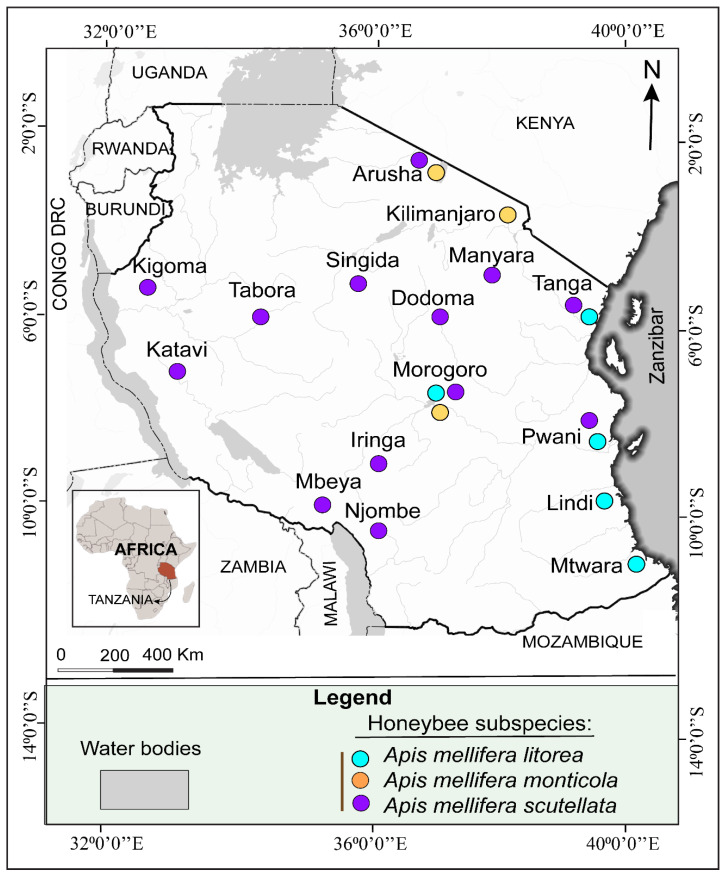
Geographical distribution of documented *A. mellifera* subspecies in Tanzania. The occurrence of *A. m. litorea* in the Morogoro Region indicates an extended range beyond coastal areas. The widespread occurrence of *A. m. scutellata* and the absence of subspecies information in island areas highlight the need for molecular studies to confirm nationwide biodiversity.

Tanzania hosts a high diversity of wild and managed bee species [13]. *Apis mellifera* subspecies was initially described in the 1930s as *A. m. adansonii* with altitudinal variations [9,14,15]. Subsequent studies in morphometric [16] and molecular identification [3] have recognized three subspecies which utilized by beekeepers [4]. These subspecies occupying diverse environments from low to high altitude areas [3,4,17,18,19]. Their geographical distribution is illustrated in Figure 1. *Apis mellifera monticola* is a large dark-colored subspecies originating from cool forests on mountain slopes, found at altitudes of 1500–3000 m on Mt. Kilimanjaro, Mt. Meru, and the Udzungwa Mountains (Figure 1). *Apis mellifera litorea* is a small yellowish subspecies identified in coastal areas, lower altitudes of the Kilombero River basin, and lowland montane forests [16].

*Apis mellifera scutellata is* a plateau-adapted subspecies found in savanna regions like Tabora, Kigoma, and Morogoro [3,4,17,18,19]. They are characterized by medium body size and yellowish coloration [18]. This prolific and highly productive plateau subspecies is found nationwide (Figure 1) and commonly managed by indigenous beekeepers [4,20]. However, *A. m. scutellata* is known for traits such as absconding in response to pest and predator attacks [21]. Its seasonal migratory swarming behavior is often linked to brood synchronization difficulties during periods of food scarcity [21,22]. The presence of *A. m. monticola* and *A. m. litorea* beyond their native ranges (Figure 1) indicates wider distribution. While morphometric methods have been widely used to identify honeybee subspecies in Tanzania, their accuracy is limited by phenotypic plasticity and hybridization. Molecular identification offers higher taxonomic resolution for accurate subspecies discrimination, gene flow detection, and genetic diversity assessment. Expanded molecular studies on the biology and biodiversity of *A. mellifera* subspecies can improve their management and conservation efficiency.

### 3.2. Honeybee Habitat and Forage Resources

Tanzania is renowned for its rich biodiversity [23]. Its forests and woodlands extend over 48.1 million hectares, covering about 55% of the country’s total land area [24]. This country harbors an estimated 9.2 million honeybee colonies [5]. Diverse wild plant species in miombo woodlands, mangrove ecosystems, and acacia-dominated vegetation provide abundant and seasonally varied forage resources for honeybees [25]. Over 95% of beekeeping activities occur in savanna forests within major beekeeping zones. These forests are characterized by wet and dry miombo woodlands that are widespread in western regions like Tabora, Katavi, and Kigoma [26]. Optimal utilization of forage and habitat resources could theoretically enable annual maximum production of 138,000 tonnes of honey and 9200 tonnes of beeswax [5]. Although the current production remains far below this potential due to management, environmental, and socio-economic constraints [7], equipping beekeepers with better tools, training, and support for value addition and market access can help minimize this gap.

## 4. Beekeeping Practices

### 4.1. Traditional Practices

Honey hunting and gathering were among the earliest beekeeping practices in Tanzania [19]. It continues to be practiced by a few hunter-gatherer ethnic groups such as the Hadzabe, Sonjo, Ndorobo, Daatoga, and Maasai [27,28]. Traditional beekeeping emerged in the post-honey-hunting era with increased understanding of bee behavior and environmental challenges leading to a decline in hunting [29]. More than 75% of indigenous beekeepers in Tanzania rely on traditional practices [24] with varied reliance across agroecological zones [11,30]. For instance, approximately 60% of beekeepers in the Eastern zone, 80% in the Western zone, and 80% in Zanzibar depend on these practices [26,31,32].

**Figure 2:**
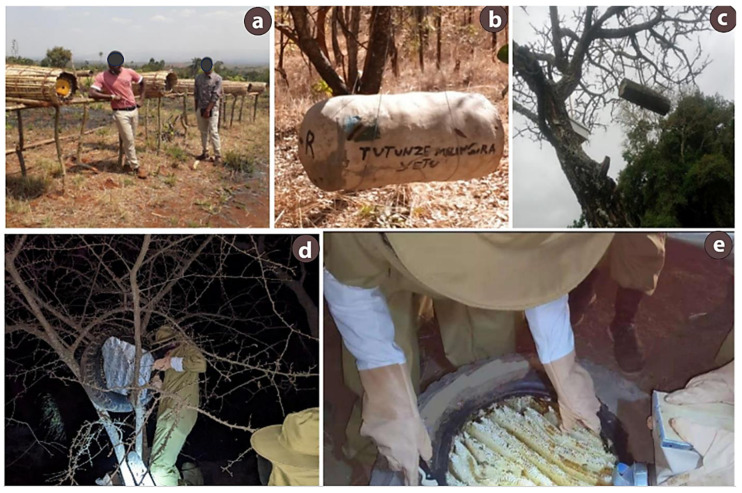
Traditional beekeeping practices in Tanzania: (**a**) an apiary with hives made from cow dung, dry grasses, and sticks in the Western zone; (**b**) a concrete hive suspended from tree branches to deter honey badgers in the Southern highlands; (**c**) log hives hung on trees in the Coastal Zone; and (**d**,**e**) nighttime honey harvesting from car tire hives managed by Maasai communities in the Northern Zone.

Traditional beekeeping involve hives crafted from tree bark, logs, dry grasses, old car tires, concrete, pottery, straw, and bamboo (Figure 2) [9,30,33]. Local knowledge guides hive placement and harvest timing. Hives are hung on trees to trap wild colonies, and honey is often harvested at night (Figure 2c–e) [5,22,34]. Placing hives high on tree branches protects them from fire and predators [9,21,30]. Many beekeepers practice api-agroforestry by suspending hives from tree branches to deter theft and predators [35,36]. Traditional hives constitute approximately 90% of all beehives in the country [24]. These hives have crafted using indigenous beekeepers’ knowledge of estimating natural nest cavities [4]. An estimated 1,506,345 traditional hives (logs and bark) and 23,650 improved hives (top-bar or Langstroth) have been documented nationwide [10].

Hive stocking typically involves trapping honeybee colonies using bait materials like beeswax, cow dung, leaves, and grasses [7]. Reliance on traditional fixed-comb hives limits colony management, damages hive resources, reduces honey yields and profitability [7,8,37]. For instance, beekeepers using top-bar hives in central zone achieved higher colony occupancy (84.7%) and greater annual yields (22.2 kg per hive) compared to those using log hives (65.43% and 7.98 kg, respectively) in 2025 [37]. Replacing fixed-comb hives with movable-comb systems and adopting semi-modern practices can significantly improve productivity and sustainability. These practices support api-agroforestry, incorporate local knowledge for swarm capture, and protect hives from wildfires and predators.

### 4.2. Commercialization and Modern Practices

**Figure 3:**
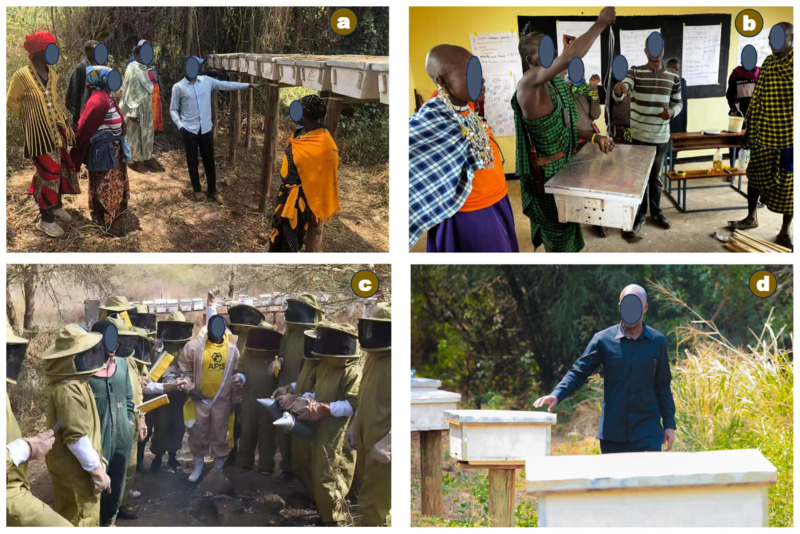
Commercialization of beekeeping practices in Tanzania: materials and technical support from (**a**) OIKOS East Africa and (**b**) WWF Tanzania encouraging Maasai youth and women’s participation in conservation-focused beekeeping; (**c**) training and consultancy by Apis Bee Company; and (**d**) TAFORI’s nationwide survey of beekeepers’ knowledge and hive types.

Beekeeping in Tanzania is predominantly practiced by small-scale farmers using traditional fixed-comb hives. This reflects a technological gap that limits productivity, harvesting efficiency, product quality, and access to high-value markets [38]. Improved beehives are either imported or locally manufactured based on dimensions suited to western honeybees [39]. Modernization initiatives are promoted through collaborations among government institutions, the private sector, and non-governmental organizations (Figure 3) [20,40]. These efforts largely focus on material provision and technical support. For example, adoption of movable-comb hives has resulted in higher honey yields and incomes in regions such as Tabora, Katavi, and Singida compared with fixed-comb systems [26,37]. Despite interventions like technology dissemination, cooperative formation, and contract farming, the sector’s commercialization potential remains largely unrealized [8,38].

Although improved hives facilitate colony inspection and management, indigenous beekeepers often reject them due to technical challenges and cultural factors [7,8,38]. Beekeepers in the western zone reported an average annual income loss of USD 1131.5 due to frequent colony absconding in improved hives [39]. This problem is partly attributed to adopting European hive designs that mismatch local practices and the behavioral and ecological traits of local subspecies [4,39]. Recent studies indicate the natural bee space of northern Tanzanian honeybees (11.82 ± 0.36 mm) exceeds the 7.5 ± 1.5 mm typical for European subspecies [39]. In Udzungwa Mountain areas, natural swarms preferentially occupy hive cavities with volumes of 12,000 to 440,000 cm^3^, entrance areas of 8.25 to 16.5 cm^2^ oriented toward sunrise or sunset, and heights of 2–19 m above ground [4]. These characteristics differ markedly from standardized Langstroth (45,402 cm^3^) and top-bar hives (67,804 cm^3^) [4,39]. Such mismatches contribute to low swarm capture, increased swarming and absconding, and cross-combing, which limit the benefits of movable-comb systems [4,39]. Combining traditional practices with modern scientific knowledge can improve the effectiveness and sustainability of commercial beekeeping. Effective technology transfer can be improved by aligning it with the behavioral and ecological needs of local honeybee populations.

### 4.3. Apiaries and Bee Reserves

Apiaries have been established in bee reserves, forest reserves, farmlands, and wildlife-protected areas [41]. Over 1533 apiaries are managed by government agencies, private institutions, NGOs, and individual beekeepers [42]. National Beekeeping Act No. 15 of 2002 [43] mandates sufficient forest allocation for honeybee development [42]. Under the Act, bee reserves have been designated to promote sustainable beekeeping across the country [5,40]. Tanzania Forestry Services Agency (TFS) has established nine official bee reserves and one private reserve that covers over 18,472 hectares [10]. TFS manages 205 apiaries with 14,292 beehives nationwide and allocated approximately 1.2 million hectares of forest for apiaries and bee reserves [10]. Nonetheless, the Village Land Act [44] empowers local councils to manage communal resources like Village bee reserves [40,43]. Community-based forest management through apiaries and bee reserves has enhanced conservation of honeybee habitats and forage resources [42]. These initiatives have improved household economic resilience and contributed to poverty reduction through income from honey and beeswax [10,40]. Benefits are evident in rural communities surrounding reserved forests in central, western, southern highlands, and northern zones. In these regions, TFS produced 69.09 tonnes of honey, 3.4 tonnes of beeswax, and 37 kg of pollen between 2018 and 2020 [10]. Implementing community-based beekeeping can improve the sustainable management of apiaries and bee reserves.

### 4.4. Honeybee Pests, Parasites, and Predators

**Figure 4:**
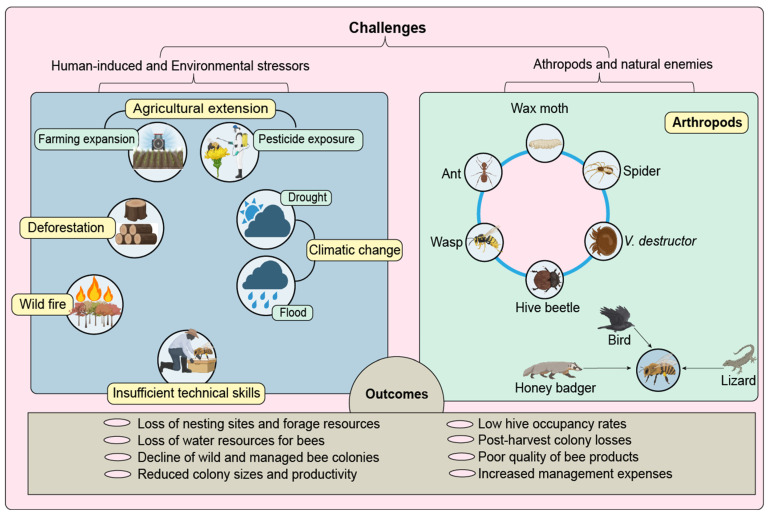
Anthropogenic (human-induced) factors, environmental stressors, arthropod pests and parasites, and natural enemies have been identified as challenges to beekeeping management across various regions of the country.

Beekeepers frequently report honey badger (*Mellivora capensis*) as a significant predator that destroys hives [45]. The predator’s reports span across southern highlands [46], northern [47], and western [48] zones. Arthropods like ants, bee lice, wasps, wax moths, and hive beetles are considered major pests [48]. Meanwhile, birds, spiders, lizards, and honey badgers are primary predators that affect beekeeping operations [20,48]. Honeybee pests, parasites, and predators are categorized as natural honeybee enemies, as shown in Figure 4.

In recent decades, studies have confirmed the presence of *Varroa destructor* (Mesostigmata: Varroidae) [20,49] with altitudinal variation in infestation levels across the country [49,50]. Honeybee colonies appear largely unaffected by mite infestations, likely due to adaptation and inherent ability to coexist with the parasite [20]. These characteristics make them an important genetic resource for developing mite-resistant strains, potentially lowering acaricide use, boosting colony survival, and increasing productivity. Further research is needed to assess the broader impacts of pests, parasites, and predators on honeybee health and productivity.

### 4.5. Pesticide Exposure

Tanzanian government encourages apiary establishment on agricultural land to enhance bee product yields and pollination services, supporting crop production and food security [5]. For example, honeybee colonies have been introduced to sunflower farms in southern [51] and western [52] zones to improve yields. The honeybee-pollinated watermelons in the northern zone were found to produce superior-quality fruits [53]. However, studies from the central zone have documented insecticides, fungicides, and herbicides contamination in honeybees and hive products [54].

Agrochemical applications in southern highlands reduced forage quality by changing nectar and pollen composition, resulting in decreased honeybee species richness, diversity, and population density [55]. Analysis of beeswax samples from the central zone detected 252 pesticide residues at an average concentration of 0.03 mg/kg, with lambda-cyhalothrin most prevalent [54]. Although there are no standardized maximum residue limits (MRLs) for beeswax, reference values such as the EU’s default limit of ≤0.01 mg/kg for unspecified residues in honey provide a useful point of comparison [54,55]. Residues in wax are concerning due to their potential chronic and sublethal effects on brood development, behavior, immunity, and colony performance [55]. National beekeeping guidelines advise maintaining a precautionary buffer zone of at least 7 km between apiaries and intensively cultivated areas [56]. This recommendation has been tailored to local conditions, taking into account landscape characteristics and prevailing farming practices. Absence of systematic field data highlights the need for thorough monitoring and research to measure exposure levels, assess residues against international standards, and determine their impact on honeybee health and product safety.

## 5. Bee Product Industry

### 5.1. Production and Processing of Bee Products

Tanzania is endowed with warm temperatures, adequate sunlight, and well-distributed rainfall that create a conducive climate for bee product production [57]. Honey and beeswax are produced in significant quantities [12]. In 2021, Tanzania produced about 31,179 tonnes of honey and 1865 tonnes of beeswax, ranking it second in Africa and tenth globally for honey production [7,58]. This production primarily comes from ten high-potential regions like Tabora, Katavi, Kigoma, Shinyanga, Geita, Kagera, Rukwa, Songwe, Mbeya, and Singida [59]. National average productivity under improved management is annually estimated at 15 kg of honey and 2 kg of beeswax per hive [24,59,60]. Nevertheless, productivity varies widely with management intensity, floral resource availability, and colony performance [4,61,62].

Furthermore, other products like bee pollen, royal jelly, bee venom, and propolis are produced in minute quantities [63]. These products remain underexploited and undocumented within the country [24]. In 2019, the government established five modern honey processing factories in high-potential districts, including Sikonge, Mlele, Bukombe, and Kibondo [10,59,64]. However, the majority of indigenous beekeepers still use traditional methods for processing honey and beeswax [65,66]. For example, beeswax is commonly processed using traditional methods such as the Tanganyika technique in the northeastern and coastal zones [22]. Tax exemptions on imported and locally manufactured beekeeping equipment can enable beekeepers to enhance the quality and yield of bee products.

### 5.2. Bee Products Quality and Properties

**Figure 5:**
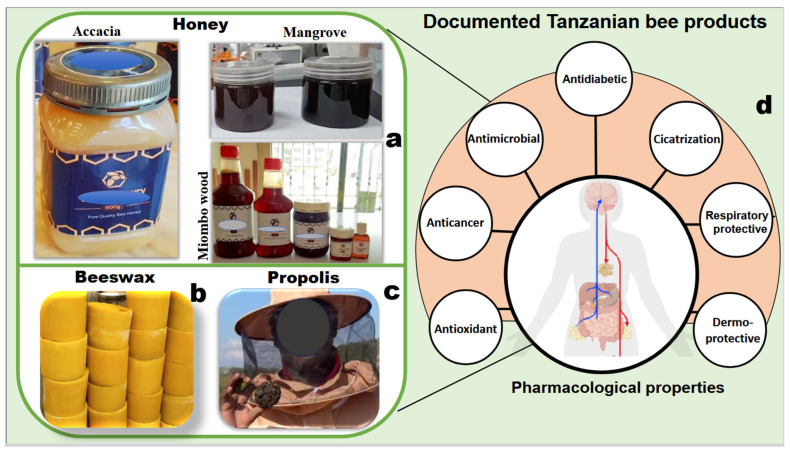
Visual characteristics of bee products derived from different vegetation types in Tanzania: (**a**) variation in honey color from acacia, miombo woodlands, and mangrove forests, including stingless bee honey from miombo; (**b**) beeswax from miombo woodlands of western and southern highlands; (**c**) propolis from mangrove-associated marine ecosystems; and (**d**) pharmacological properties identified across Tanzanian bee products. In panel (**d**), blue indicates the nervous and digestive systems, whereas red indicates the respiratory and endocrine systems.

Tanzania has high potential for producing safe and quality bee products due to predominantly wild-based and chemical-free beekeeping in miombo woodlands [67]. Figure 5 illustrates visual characteristics of honey, beeswax, and propolis from various vegetation types. Majority of beekeepers produce honey and beeswax that comply with local and export quality standards [68]. This adherence is promoted through continuous training sessions and enhanced use of improved beekeeping equipment [69]. Various honey samples from selected regions have water content below 20%, sugar levels above 65%, ash content below 0.5%, acidity below 40 meq/kg, and hydroxymethylfurfural (HMF) levels below 40 mg/kg [68,70,71].

Nonetheless, high-quality beeswax is commonly produced in the widely distributed miombo woodlands (Figure 5b) [72]. The country’s propolis is rich in bioactive compounds, making it valuable for local pharmacological applications (Figure 5c,d) [63,73,74]. Expanded solution-based scientific research can help address existing knowledge gaps related to the production, properties, quality, and applications of bee products beyond honey.

### 5.3. Consumption of Bee Products

Tanzanian honey exhibits a distinctive phytochemical profile reflecting diverse vegetation and mineral-rich environments [68,75]. Inductively coupled plasma–mass spectrometry analysis (ICP-MS) of honey from various agroecological zones revealed significant concentrations of manganese, iron, zinc, and copper [76]. Dark-colored miombo woodland honey (Figure 5a) contains essential minerals and vitamins that confer medicinal properties and contribute to its global demand [68,70]. It exhibits strong antimicrobial activity against pathogens like *Staphylococcus aureus*, *Escherichia coli*, *Salmonella typhi*, and *Candida albicans* [77,78]. Potent antioxidant activity and high phenolic content of miombo wood honey contribute to its effectiveness against both communicable and non-communicable diseases [79].

Propolis from different regions shows effective antimicrobial, antidiabetic, and anticancer potential [63,73]. It is traditionally used to treat skin, wounds, and respiratory infections (Figure 5d) [74]. Many findings on the uses of bee products are derived from in vitro studies that offer limited direct relevance to human health. These studies are also limited by small sample sizes, diverse sources, and the absence of standardized clinical trials. Well-designed clinical investigations can help validate the specific therapeutic effects of honey and propolis.

## 6. Beekeeping-Based Conservation Initiatives

**Figure 6:**
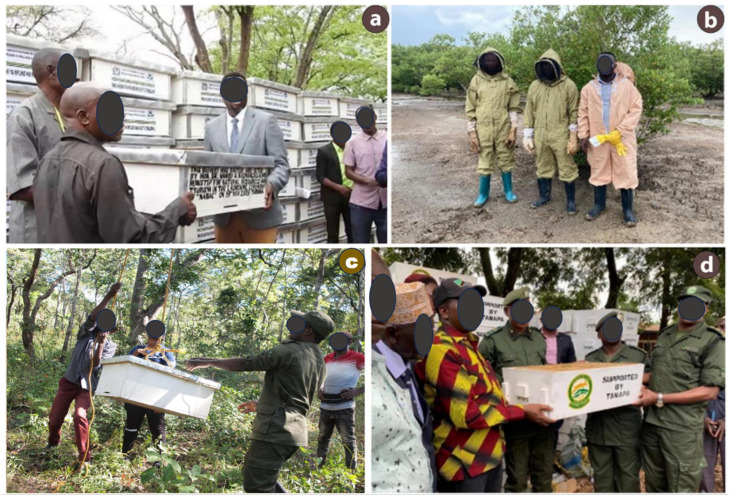
Utilization of beekeeping resources for natural resources conservation in Tanzania: (**a**) protection of forest ecosystems through enhanced community-based beekeeping practices in the Western zone; (**b**) application of beekeeping for restoration and conservation of mangroves ecosystem along coastal mainland and island areas; (**c**) TFS-supported rural beekeeping initiatives mitigating human–elephant conflicts around Nyerere National Park; and (**d**) community-based beekeeping programs at Gombe National Park strengthening wildlife management through collaboration between park authorities (TANAPA) and adjacent local communities.

Beekeeping supports biodiversity conservation in Tanzania by protecting forest ecosystems, sustaining wildlife, and promoting environmentally responsible resource management (Figure 6). The Forestry and Beekeeping Division (FBD) promotes landscape restoration, ensures fodder plant availability, and encourages api-agroforestry and reforestation [9]. Since the 1990s, communities have been provided with financial incentives to engage in environmental conservation through beekeeping incorporated into forest and wildlife management programs [80,81].

Historically, beekeeping has contributed to forests and biodiversity conservation through community-based forest initiatives and establishment of bee reserves and beekeeping zones [12]. Association for the Development of Protected Areas (ADAP) promoted beekeeping as a sustainable alternative to destructive practices for communities near miombo woodlands. This was achieved by providing training, capacity building, improved techniques, and micro-credit in western Tanzania [40]. OIKOS East Africa and WWF Tanzania launched conservation projects to restore degraded land through community-based forest reserves in northern zone. A key component involved promoting small- to medium-scale beekeeping among Village Game Scouts (VGS) and Village Cooperative Society (VCS) groups in Maasai communities (Figure 3a,b). In addition, beekeeper groups and TFS actively work to prevent bushfires and protect forests from illegal logging and charcoal production through beekeeping activities nationwide (Figure 6a,b) [35].

Beekeeping initiatives in communities surrounding national parks and game reserves such as Udzungwa, Ruaha, Kitulo, Serengeti, Ngorongoro, Nyerere, Gombe, Ugalla, and Mkomazi have contributed to reduced human–wildlife conflicts [4,31,41]. These initiatives have also strengthened collaboration and trust between conservation authorities and local communities (Figure 6c,d) [31]. Beehive fences have successfully deterred large wildlife like elephants and gorillas from the communities around Nyerere and Gombe National Parks (Figure 6c). Quantitative studies on conservation outcomes of beekeeping such as forest cover retention and reduced poaching, together with assessments of the long-term sustainability of related livelihood gains, can substantiate the proposed theoretical benefits.

## 7. Challenges Facing Beekeeping Industry

Beekeeping industry faces various constraints arising from both anthropogenic and natural factors (Figure 4) that can be grouped into four interrelated categories.

(i) Loss of habitat and forage resources. Agricultural expansion, charcoal production, and some cultural practices continue to drive deforestation and forest degradation [82,83]. These practices lead to the loss of honeybee nesting sites and floral resources [82]. In addition, prolonged droughts reduced colony productivity and survival across regions by limiting the availability of nectar, pollen, and water [48].
(ii) Honeybee colony losses. Declines in both wild and managed colonies are associated with environmental stressors. Regions such as Manyara, Morogoro, Katavi, and Tabora have experienced seasonal migration, absconding, pest and predator pressure, cross-combing, and post-harvest disturbances [4,39,48]. Studies have revealed a nationwide reduction in swarm availability, low hive occupancy, small colony sizes, and post-harvest colony losses [7,62]. Nonetheless, pathogens such as European foulbrood (*Melissococcus plutonius*), nosemosis (*Nosema apis* and *N. ceranae*), and honeybee viruses have been reported in neighboring countries [84]. Their occurrence within Tanzania remains unconfirmed, and current evidence indicates that they have not yet been formally documented [85].
(iii) Low technical capacity and technological development. Limited adoption of modern technologies constrains effective exploitation of Tanzania’s apicultural potential [7,61]. Traditional fixed-comb hives vary in size and structure which limit systematic colony management and compatibility with modern honey extraction and processing equipment [12,86]. Most beekeepers operate at a small scale with limited technical training, and inadequate facilities and skills contribute to low product quality and weak colony management [11,60].
(iv) Governance and institutional capacity. The sector encounters institutional challenges, such as restricted access to organic certification and inadequate intellectual property safeguards [87]. Insufficient scientific and technical expertise limits progress in standardization, value addition, and product diversification [24]. Beekeeping interventions are hampered by low awareness of supportive policies, guidelines, and development programs [61]. Reduced operational effectiveness is compounded by weakly organized cooperatives and community groups [86]. Coordinated policy enforcement, legal and regulatory reforms, strengthened research and extension services, and enhanced stakeholder collaboration can help develop a resilient beekeeping sector.

## 8. Future Directions

Beekeeping sector has considerable potential to contribute to economic growth, food security, environmental sustainability, and natural resource conservation in Tanzania. Realizing this potential requires prioritizing four key research and policy interventions.

1. Research on honeybee health, productivity, and biodiversity. Additional in-depth research is needed to evaluate health, ecology, and genetic diversity of Tanzania’s native honeybee subspecies. The results will support development of region-specific hive designs and management practices to minimize colony losses due to absconding, pests, diseases, and habitat change. Establishing well-equipped research centers can facilitate molecular programs to support the selective breeding and biodiversity confirmation of local honeybee subspecies. These centers will also facilitate the development of diverse bee products such as pollen, venom, propolis, and royal jelly. Strengthening honeybee health will enhance colony survival, improve pollination services, and support opportunities in apitherapy, apitourism, and conservation.
2. Beekeeping commercialization through capacity building and technology transfer. Sector transformation depends on equipping beekeepers with appropriate skills and technologies. Strengthening training institutions and extension services will promote improved, locally adapted practices in production, quality control, and value addition. Promoting semi-modern practices such as movable-comb hives adapted to local honeybee ecology and behavior, and combined with indigenous knowledge can enhance colony management and reduce losses. For instance, modifying traditional hives into top-bar designs that align with local comb dimensions improves swarm capture, reduces cross-combing, facilitates management, and supports natural bee behavior.
3. Strengthening governance, coordination, and data systems. Establishing a national association of beekeeping professionals will foster collaboration among researchers, practitioners, investors, and policymakers. Strengthened partnerships at national and international levels can improve market access, certification, value addition, and innovation. Better registration systems and monitoring of beekeepers and colonies, along with improved inter-sectoral collaboration (agriculture, forestry, wildlife, health, manufacturing) can support evidence-based planning. Advanced digital data systems can enhance information sharing among indigenous beekeepers, government agencies, development partners, investors, and consumers.
4. Evidence-based assessment of cross-sectoral contributions. The assertion that beekeeping contributes to livelihoods, biodiversity, agricultural productivity, ecotourism, and community health is not yet supported by sufficient quantitative evidence in the available literature. Expanded quantitative studies will strengthen the practical relevance of the sector. Integrated socio-economic and conservation research that considers local honeybee behavior, ecology, and indigenous knowledge can improve the effectiveness and sustainability of management practices. In addition, coordinated investigations of bee product properties, value addition, and applications through field and clinical trials can validate medicinal uses and support diversification beyond honey and beeswax.

Sustainable growth of Tanzania’s beekeeping sector depends on evidence-based policies and strong institutional coordination. Integrating indigenous knowledge with modern technologies is also essential. Strategic investments in honeybee health research, capacity building, governance, and cross-sector studies will optimize apicultural resources. This approach positions beekeeping as a key contributor to livelihoods, biodiversity conservation, and sustainable green growth.

## Data Availability

The original contributions presented in this study are included in the article Further inquiries can be directed to the corresponding author.

## Abbreviations

The following abbreviations are used in this manuscript:

ADAP	Association for the Development of Protected Areas
BTI	Beekeeping Training Institute, Tabora
MNRT	Ministry of Natural Resources and Tourism
NBP	National Beekeeping Policy
NGO	National Beekeeping Policy
TAFORI	Tanzania Forest Research Institute
TANAPA	Tanzania National Parks Authority
TFS	Tanzania Forest Services Agency
URT	United Republic of Tanzania
VCS	Village Cooperative Society
VGS	Village Game Scouts
WWF	World Wildlife Fund
